# Caveolin-1 promotes tumor cell proliferation and vasculogenic mimicry formation in human glioma

**DOI:** 10.1590/1414-431X2020e10653

**Published:** 2021-07-16

**Authors:** Wenli Chen, Xing Cheng, Xiaobo Wang, Wenjie Hu, Jinshan Wang, Chuangxin Liao

**Affiliations:** 1 Department of Neurosurgery, The First Affiliated Hospital of Sun Yat-Sen University, Guangzhou, China; 2 Department of Spine Surgery, The First Affiliated Hospital of Sun Yat-Sen University, Guangzhou, China; 3 Laboratory of Ocular Neurovascular Biology, State Key Laboratory of Ophthalmology, Zhongshan Ophthalmic Center, Sun Yat-Sen University, Guangzhou, China

**Keywords:** Glioma, Caveolin-1, Vasculogenic mimicry, Akt pathway, Hypoxia-inducible factor 1α

## Abstract

Vasculogenic mimicry (VM) plays an important role in human glioma progression and resistance to antiangiogenic therapy as a compensatory neovascularization mechanism in malignant tumors. Caveolin-1 (Cav-1) has been found to contribute to VM formation. However, it remains largely unknown whether Cav-1 expression correlates with VM in glioma. In this study, we examined CAV-1 expression levels and VM in human glioma cell lines and in 94 human gliomas with different grades of malignancy, and present Cox proportional hazards regression. The molecular role of Cav-1 in glioma cells was investigated using quantitative polymerase chain reaction (qRT-PCR) assays, western blotting, CCK-8 assays, and tubule formation assays. Cav-1 expression and VM formation were positively correlated with each other and both were closely associated with glioma development and progression as evidenced by the presence of cystic tumor, shortened survival time, and advanced-stage glioma in glioma patients with Cav-1 overexpression/increased VM formation. Cav-1 promoted U251 glioma cell proliferation and VM formation in a Matrigel-based 3D culture model. VM-associated factors including hypoxia-inducible factor 1α (HIF-1α) and p-Akt was significantly elevated by Cav-1 overexpression but suppressed by siCav-1 in U251 cells. Collectively, our study identified Cav-1 as an important regulator of glioma cell proliferation and VM formation, contributing to glioma development and progression.

## Introduction

Glioma is the most common malignant primary tumor in the central nervous system, posing a considerable threat to human health (1). Despite the recent advances in standard treatment options, including surgery, radiation therapy, and chemotherapy, the median overall survival in patients with glioma remains as low as 15 months (2), which highlights the necessity of developing effective therapeutic strategies against glioma. Angiogenesis is a complex process involving formation of new blood vessels that can provide tumor tissues with oxygen and nutrients, thus playing a critical role in glioma growth and metastasis (3). Targeting angiogenesis has been an US Food and Drug Administration-approved therapeutic strategy for glioma treatment since 2004 (4). However, drug resistance or high rates of relapse greatly limit the clinical application of currently available angiogenesis inhibitors in glioma therapy (5). Therefore, identifying new therapeutic targets is urgently required for developing potential drugs against glioma.

Vasculogenic mimicry (VM) was first reported in 1999 by Maniotis et al. (6) as a non-endothelium-dependent vasculature composed of tumor cells and a basement membrane that allows blood plasma and red blood cells to flow in. VM serves as an irrigation system for tumor cells to meet their increasing metabolic and nutrient demands. Existence of VM can be evidenced by periodic acid-Schiff (PAS) staining due to a high abundance of laminin, proteoglycans, heparan sulfate, and collagens in the extracellular matrix of tumor cells (7,8). CD31^-^/PAS^+^ staining is regarded as the golden standard for tumor cell-lined VM (6,9–12). Previous studies demonstrated that VM is correlated with the degree of tumor malignancy and prognosis in patients with glioma (7,8). Hypoxia resulting from antiangiogenic therapy in glioma may induce VM (compensatory neovascularization) to counteract the hypoxic environment within the tumor, leading to resistance to antiangiogenic therapy (13). Thus, identifying the genes and signaling pathways implicated in VM formation is needed to develop effective therapeutic options for glioma.

Human caveolin-1 (Cav-1), a principle structural protein of caveolae, has been shown to act as either a tumor promoter or suppressor depending on the tissue type (14–17). In glioma, Cav-1 exhibits a tumor suppressive role both *in vitro* and *in vivo* through inhibiting TGFβ/SMAD pathway or activating apoptosis. On the other hand, Cav-1 was also found to be upregulated proportionally to glioma grades, which suggests a promotive role of Cav-1 in glioma progression (18–20). Therefore, the function of Cav-1 in glioma development remains controversial. Stenzel et al. (21) has reported that Cav-1 expression is correlated with PI3K activity and VM in uveal melanoma, suggesting that Cav-1 may induce VM formation through the PI3K/Akt signaling cascade. In hepatocellular carcinoma and renal cell carcinoma, Cav-1 is induced by hypoxia via hypoxia-inducible factor 1α (HIF-1α), suggesting a possible role of Cav-1 in tumor angiogenesis (22,23). However, the expression pattern of Cav-1 and the relationship between Cav-1 and VM in glioma remain unclear.

In this study, we examined the expression of Cav-1 and VM formation in glioma tissues. The correlations between Cav-1 and VM in glioma patients as well as between Cav-1 expression/VM formation and the clinicopathological characteristics were determined. The effects of Cav-1 overexpression and knockdown on glioma cell proliferation and VM formation were also investigated.

## Material and Methods

### Patients and samples

Tissue samples were obtained from 94 patients with primary glioma undergoing surgical resection at the Department of Neurosurgery, The First Affiliated Hospital of Sun Yat-Sen University, Guangdong, China from January 2010 to July 2014. No patient received chemotherapy or radiotherapy prior to surgery. Final diagnosis was confirmed by two independent pathologists and graded according to the 2016 World Health Organization grading system for central nervous system tumors. Four normal brain tissue samples were collected from patients with hernia during surgical decompression. All tissue samples were fixed in 4% neutral buffered formaldehyde at 4°C followed by paraffin embedding. The inclusion of patients in this study was only dependent on the availability of tumor materials and clinical follow-up data. The follow-ups were terminated in July 2017. This study was approved by the Ethics Committee of Sun Yat-Sen University and was in accordance with the Declaration of Helsinki (Ethics committee approval number, 2021150). Written informed consent was obtained from each patient.

### Immunohistochemical (IHC) staining

Paraffin-embedded tissue sections (4-µm thick) were deparaffinized and rehydrated followed by IHC staining for glial fibrillary acidic protein (GFAP), Cav-1, or CD31. For antigen retrieval, the sections were heated in 1 mM EDTA (pH 8.0) for 15 min. The sections were then blocked with goat serum followed by incubation with anti-GFAP (Abcam, USA), anti-Cav-1 (Abcam), or anti-CD31 (Abcam) at 4°C overnight and with horseradish peroxidase (HRP)-conjugated secondary antibody (Abcam) at room temperature for 30 min. Normal IgG was used as a negative control. Finally, the sections were stained with diaminobenzidine and the nuclei were counterstained with hematoxylin. An Olympus IX81 fluorescence microscope (Olympus, Japan) was used for visualization and brown staining was considered positive. Immunoreactivity of GFAP, Cav-1, and CD31 in glioma tissues was quantified by the McCarty's H-score system, which contains both the intensity of the specific staining and the percentage of positive cells. The relative intensity of specific staining was defined as not present (0), weak but detectable above control (1+), distinct (2+), and very strong (3+). The final score was the sum of the percentage of positive cells multiplied by the relative intensity of specific staining. The H-score analysis was carried out independently by two experienced pathologists in a blinded manner. A third pathologist would review the score when there was an inconsistency between the two pathologists. The Cav-1 expression levels in glioma tissues were categorized as low expression or high expression in relation to the mean value. Representative images (magnification×400) were acquired using an XDS-100 Caikang microscope (Caikang, China).

### CD31/PAS double staining

Following IHC staining for CD31, the sections were exposed to 1% sodium periodate for 10 min, rinsed with distilled water for 5 min, and then incubated with PAS in the dark at 37°C for 15 min. The sections were then counterstained with hematoxylin and the results were visualized at 400× magnification using an Olympus IX81 microscope. CD31 staining (brown) represents blood vessels in tissues, whereas CD31-negative/PAS-positive (light purple) staining represents the wall of the VM channels. The number of VM channels was counted in 5 randomly selected fields in each section. The 94 glioma tissues were classified into two groups according to the median number of VM channels.

### Immunolocalization of CAV-1 and CD31

The sections were stained with 4% paraformaldehyde for 15 min and treated with 0.1% Triton X-100 for 30 min. Rabbit anti-CD31 (1:200 Abcam) and rat anti-CAV-1 antibodies (1:200 Abcam) were added, followed by incubation at 4°C overnight. Subsequently, the sections were incubated with goat anti-rabbit secondary antibody (1:200 Invitrogen, USA) conjugated to Alexa Fluor 555, which fluoresces red, and anti-rat secondary antibody conjugated to Alexa Fluor 488, which fluoresces green, at 37°C; then, nuclei were counterstained with 4, 6-diamidino-2-phenylindole (DAPI). Images were captured using an inverted fluorescence microscope (Leica, DMI4000B, Germany). Colocalization efficiency of DAPI and CD31/CAV-1 was calculated through ImageJ software (NIH, USA). All experiments were conducted in triplicate. The staining results were observed and photographed under an inverted fluorescence microscope (IX51; Olympus).

### Cell culture and treatments

Human malignant glioma cell lines U251 and human umbilical vein endothelial cells (HUVECs) were obtained from the Cell Bank of Type Culture Collection of Chinese Academy of Science (CBTCCCAS, China). These cell lines were authenticated by DNA fingerprinting, isozyme detection, and cross species checks. All cell lines were maintained in Dulbecco's modified Eagle medium (DMEM; Hyclone, Cat. No. SH30023.01B, USA) supplemented with 10% fetal bovine serum (FBS; Hyclone, Cat. No. SH30087.01) at 37°C in a humidified incubator with 5% CO_2_.

### Generation of glioma cell lines with Cav-1 silencing or overexpression

Small interfering RNA (siRNA) targeting Cav-1 (siCav-1) was purchased from Sigma (China). The sequences of siCav-1 were 5′-CCCUAAACACCUCAACGAUdTdT-3′ (sense) and 5′-AUCGUUGAGGUGUUUAGGGdTdT-3′ (antisense). The sequences of negative control siRNA (siNC) were 5′-UUCUCCGAACGUGUCACGUTT-3′ (sense) and 5′-ACGUGACACGUUCGGAGAATT-3′ (antisense).

The coding sequence of human Cav-1 was amplified using the primers 5′-ccgctcgagATGTCTGGGGGCAAATACGTAG-3′ (forward) and 5′-cggggtaccTTATATTTCTTTCTGCAAGTTGATGC-3′ (reverse) and was inserted into the *XhoI* and *KpnI* sites of linearized pEGFP-C3 vector (Clontech Laboratories, USA) according to the manufacturer's instructions. The recombinant expressing vector pEGFP-C3-Cav-1 was sequenced by BGI Corporation (China) to confirm the cloned sequences.

U251 cells were seeded onto a six-well plate at a density of 5×10^5^ cells/well and grown overnight. pEGFP-C3-cav-1 or siCav-1 was transfected into U251 cells using Lipofectamine™ 2000 (Invitrogen) following the manufacturer's instructions. Empty vector pEGFP-C3 and the vector expressing siNC were used as negative controls. After 48-72 h of transfection, the mRNA level of Cav-1 was determined using quantitative real-time PCR (qPCR).

### Tubule formation assay

Tubule formation was evaluated using a Matrigel (BD Biosciences, USA)-based three-dimensional (3D) culture model. Briefly, transfected U251 cells were cultured for 48 h, and the conditioned medium was collected. Matrigel (50 μL) was added onto each well of 96-well plates and allowed to polymerize at 37°C for 30 min. Untransfected HUVECs were plated on Matrigel and incubated with the U251 cell-conditioned medium for 6 h at 37°C. The transfected U251 cells were also plated on Matrigel and incubated for 6 h at 37°C. The images of each well were captured using an Olympus BX61 phase-contrast fluorescence microscope (magnification ×100). Four randomly selected areas of vascular network meshes in each well were measured using ImageJ software. All experiments were performed in triplicate.

### RNA extraction and qPCR

Total RNA was isolated from tissues or cells using Trizol (Invitrogen) according to the manufacturer's protocol. The residual DNA was removed using DNase I (Roche, USA). RNA (2 μg) was reversely transcribed to synthesize cDNA using a M-MLV Reverse Transcriptase kit (Thermo Fisher, USA). The cDNA was amplified using SYBR Green qPCR Master mix (Thermo Fisher) in an ABI 7300 system (Applied Biosystems, USA) following the manufacturer's instructions. The relative mRNA level of Cav-1, HIF-1α, or Akt was calculated by normalization to that of 18s. The PCR primers were as follows: Cav-1, 5′-CACCTAAGCTGCACAGTTCC-3′ (forward) and 5′-GGCTGCCTCCTAATTCTTCC-3′ (reverse); 18s, 5′-CCTGGATACCGCAGCTAGGA-3′ (forward) and 5′-GCGGCGCAATACGAATGCCCC-3′ (reverse); AKT1, 5′-ATCGCTTCTTTGCCGGTATC-3′ (forward) and 5′-CTTGGTCAGGTGGTGTGATG-3′ (reverse); HIF-1α, 5′-GTGGATTACCACAGCTGA-3′ (forward) and 5′-GCTCAGTTAACTTGATCCA-3′ (reverse).

### Western blot analysis

Cell lysates were obtained from U251 cells or grounded tissue samples using RIPA buffer containing 1% phenylmethylsulfonyl fluoride. The protein concentration was determined using a BCA protein assay kit (Beyotime, USA). Equal amounts of total protein were separated in 10% SDS-polyacrylamide gels and then transferred onto polyvinylidene difluoride membranes. The membranes were blocked with 5% skim milk for 1 h followed by incubation with primary antibodies against Cav-1, Akt, p-Akt (Ser473), HIF-1α, or GAPDH (Abcam) at 4°C overnight. The membranes were then incubated with HRP-linked secondary antibodies (1:5,000; Beyotime) for 1 h. The protein bands were visualized using enhanced chemiluminescence assay (Thermo Fisher, USA) and exposed to X-ray films. The results were scanned and quantified using ImageJ software.

### Cell proliferation assay

Cell proliferation assay was carried out using cell counting kit-8 (CCK-8; Dojindo Laboratories, Japan) following the manufacturer's protocol. Briefly, U251 cells were seeded onto 96-well plates at a density of 2000 cell/well and transfected with siRNA. CCK-8 (10 μL) solution was added into each well at 0, 24, 48, or 72 h after transfection followed by incubation at 37°C for an additional 1 h. The absorbance was measured at 450 nm using a microplate reader (Bio-Rad, USA).

### Statistical analysis

Statistical analysis was performed using SPSS 22.0 (IBM, USA) or GraphPad Prism 6 (GraphPad, USA). The association between Cav-1 and VM was examined by Spearman analysis, and the differences of clinicopathological variables were analyzed with the chi-squared test. Statistical comparisons among groups were performed using Student's *t*-test and ANOVA. Data are reported as means±SE. All experiments were repeated independently at least three times. P<0.05 was considered statistically significant.

## Results

### Cav-1 expression/VM formation were correlated with glioma grade and overall survival

**Figure 1:**
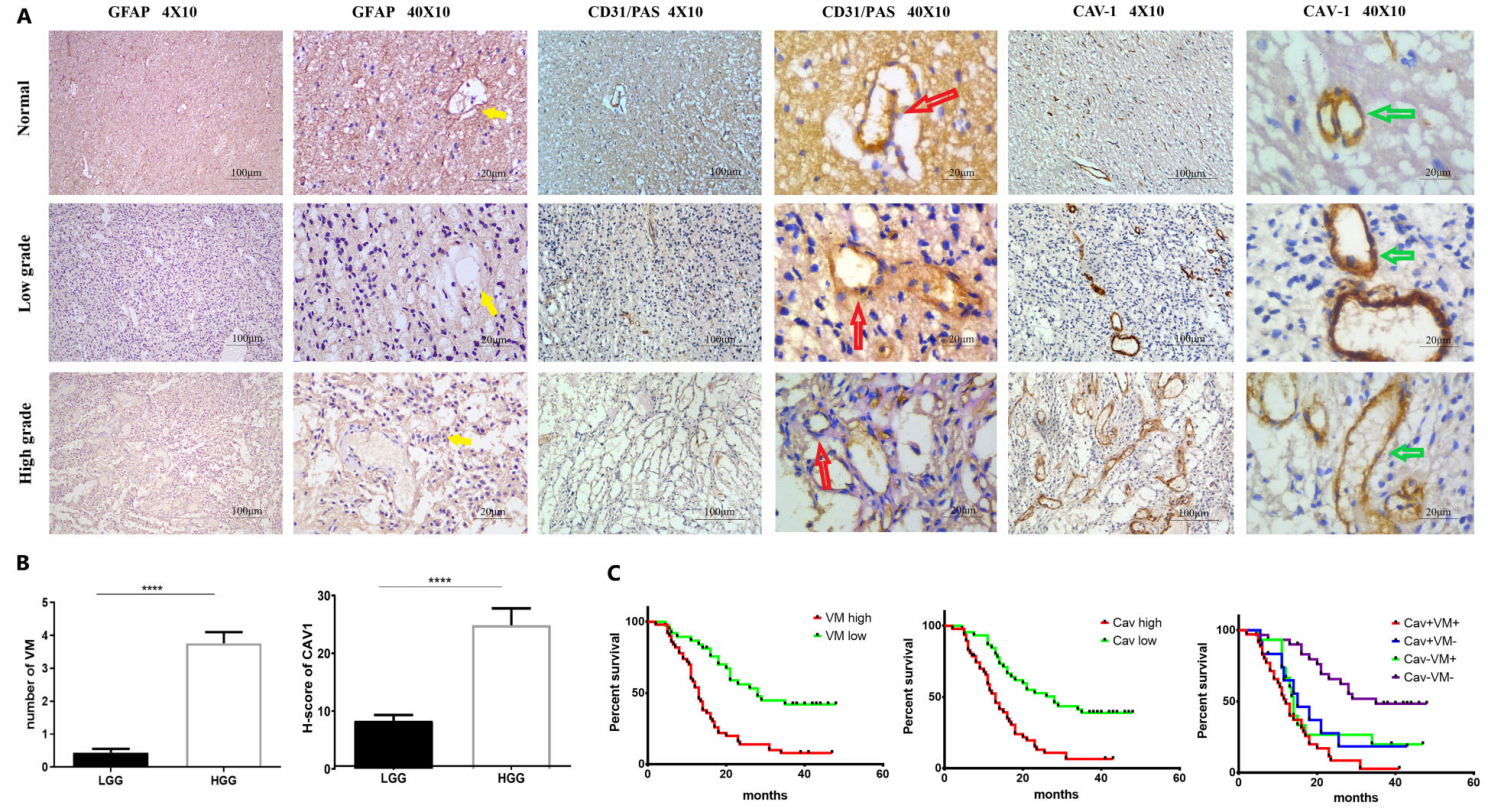
Correlation of Cav-1 expression and/or vasculogenic mimicry (VM) formation with human glioma grades and overall survival of glioma patients. **A**, Immunohistochemical staining for GFAP (brown, yellow arrow), Cav-1 (brown, green arrow), and CD31/PAS co-staining for VM channels (light purple, red arrow) in glioma specimens. Magnification 40× (scale bar 100 μm) and 400× (scale bar 20 μm). **B**, The mean number of VM channels (left) and Cav-1-positive tumor cells (right) in low-grade (LGG) (n=39) and high-grade gliomas (HGG) (n=55). Data are reported as means±SE. ****P<0.0001. **C**, Kaplan-Meier analyses of overall survival in all glioma patients with differential Cav-1 expression and/or VM formation.

**Figure 2:**
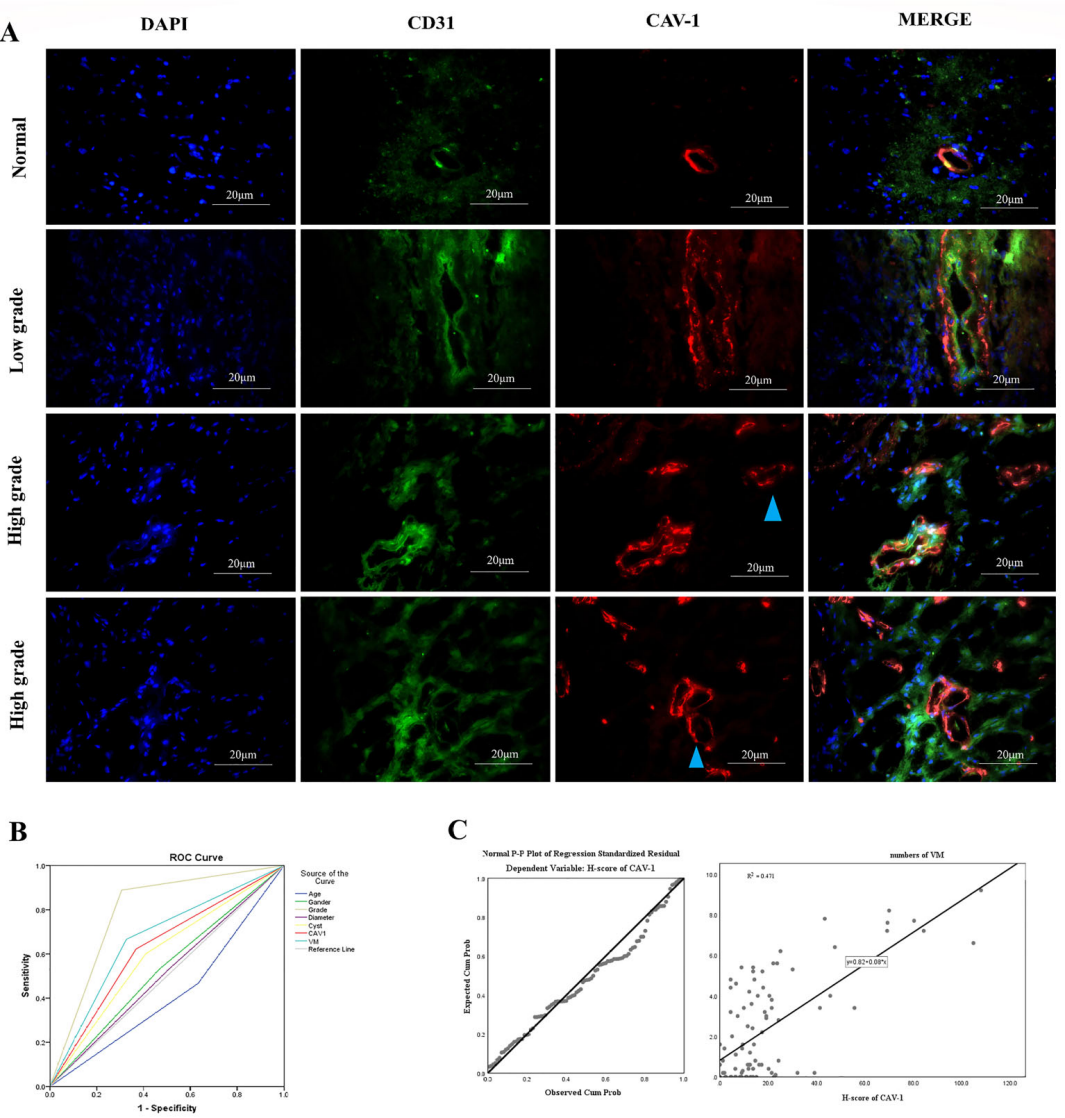
Immunolocalization and correlation between vasculogenic mimicry (VM) formation and Cav-1 expression in glioma (n=94). **A**, Cav-1 (red) and CD31 (green) expression in the blood vessels of normal tissues and low-grade gliomas and abundant Cav-1-positive (red) and CD31-negative cells around the VM-like structures (blue arrow heads) in high-grade glioma tissues (scale bar 20 μm). **B**, ROC analysis shows cause-effect relationship between factors and survival. Diagonal segments are produced by ties. **C**, Correlation between VM formation and Cav-1 expression.

As shown in Table 1 and Figure 1A and B, Cav-1 expression was significantly upregulated in high-grade gliomas (HGG) compared with low-grade gliomas (LGG), as evidenced by dramatically increased H-scores of CAV-1 in HGG compared with those in LGG (24.85±2.922 *vs* 8.318±1.016, P<0.0001). In addition, CD31/PSA co-staining showed more VM channels in HGG than LGG (3.224±0.3600 *vs* 1.108±0.2577, P<0.0001), suggesting that the VM-forming ability was remarkably enhanced in HGG compared with LGG. The abundant expression of GFAP in the cells surrounding the VM channels confirmed the cells as glial tumor cells (24,25). Immunofluorescence staining showed CD31/CAV1+ staining can differentiate VM-like structures from true blood vessels, and CAV1 may play a role in VM-like structures formed (Figure 2A). To further evaluate the potential prognostic value of Cav-1 and VM formation in glioma, we determined the association between Cav-1 expression/VM formation and glioma patient survival time using Kaplan-Meier analysis and log-rank test. As shown in Figure 1C, both high Cav-1 protein expression and VM-forming ability were significantly correlated with shortened survival of glioma patients, suggesting that upregulation of Cav-1 or VM formation was associated with poor prognosis in glioma. Interestingly, patients with Cav-1 overexpression and increased VM formation in combination had shorter survival time than those with Cav-1 overexpression or increased VM formation alone or with Cav-1 downregulation and inhibited VM formation in combination, suggesting a synergistic effect between Cav-1 expression and VM formation on glioma development and progression.

**Table 1:** Cox proportional hazards regression analysis in 94 glioblastoma patients.

Variable	Crude analysis			Adjusted analysis		
	HR	95%CI	P value	HR	95%CI	P value
Age (years)						
<60	1.0	1.0		1.0	1.0	
>60	1.16	(0.66, 2.04)	0.598	1.19	(0.67, 2.11)	0.550
Gender						
Female	1.0	1.0		1.0	1.0	
Male	1.16	(0.66, 2.04)	0.598	1.19	(0.67, 2.11)	0.550
Cystic						
No	1.0	1.0		1.0	1.0	
Yes	1.46	(0.82, 2.58)	0.196	1.38	(0.76, 2.51)	0.296
Diameter (cm)						
<5	1.0	1.0		1.0	1.0	
>5	1.02	(0.58, 1.78)	0.951	1.03	(0.58, 1.81)	0.918
Caveolin-1						
Low expression	1.0	1.0		1.0	1.0	
High expression	2.02	(1.15, 3.55)	0.015	2.05	(1.14, 3.69)	0.017^*^
VM						
Low expression	1.0	1.0		1.0	1.0	
High expression	2.61	(1.44, 4.70)	0.001	2.12	(1.13, 3.99)	0.020^$^

Adjusted by age, gender, cystic, and diameter. ^$^Adjusted by age, gender, cystic, diameter, and caveolin-1. HR: hazard risk; VM: vasculogenic mimicry.

### Cav-1 expression and VM formation in glioma patients

VM formation was positively correlated with Cav-1 expression (Figure 2B). We next sought to determine whether Cav-1 expression/VM formation is associated with clinicopathological characteristics in patients with glioma. As shown in Table 1, the presence of Cav-1 or VM was not correlated with age, sex, or tumor diameter in these 94 glioma patients. Notably, both Cav-1 expression levels and the VM-forming ability were positively correlated with the presence of cystic tumor (P=0.000 and P=0.028, respectively), WHO grades (P=0.000 for both), and survival at 16-month interval (P=0.007 and P=0.002, respectively) using multivariate analyses. These results indicated that Cav-1 expression/VM formation are associated with glioma progression and prognosis.

### Cav-1 promoted tumor cell proliferation and vascular formation in glioma

**Figure 3:**
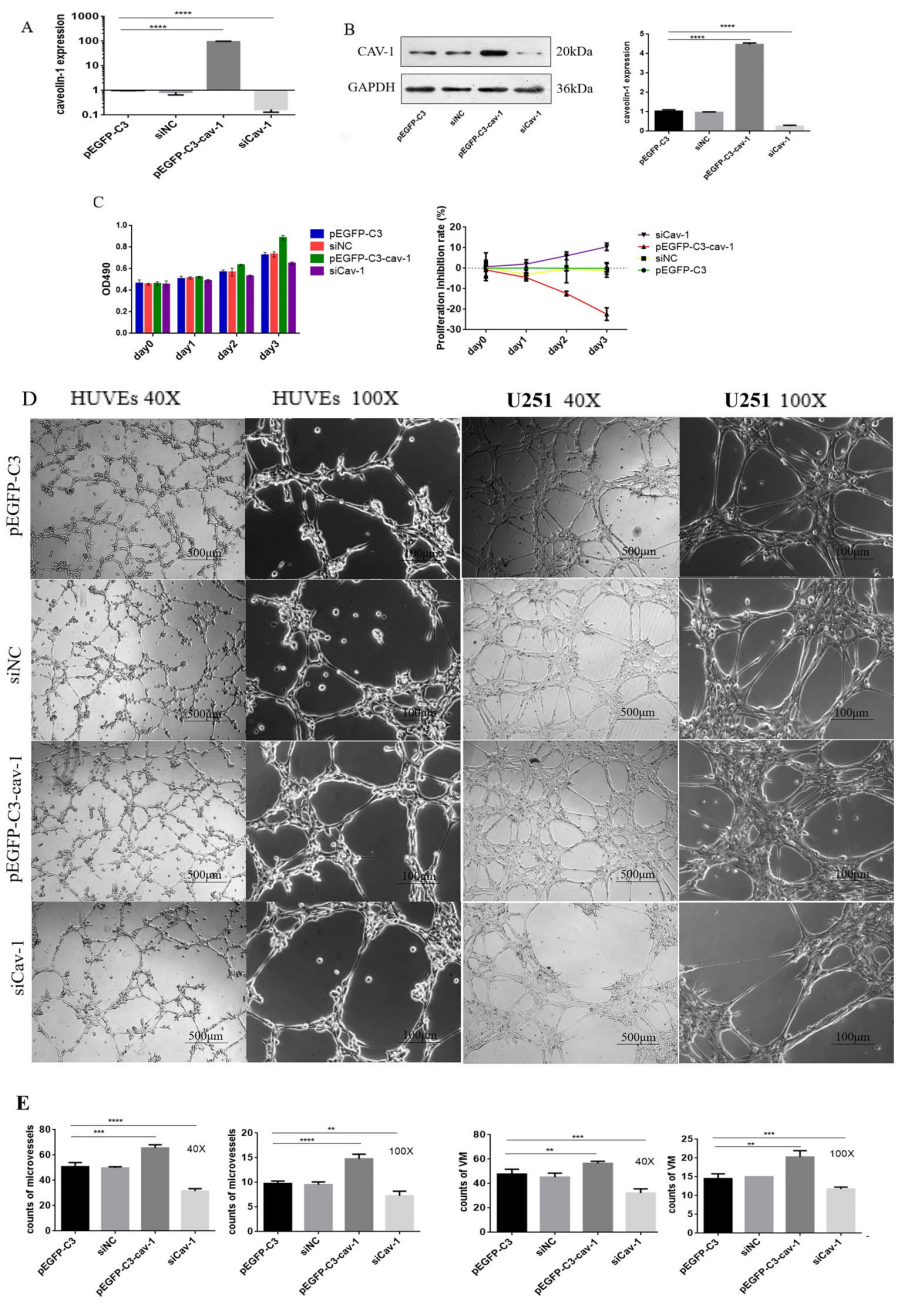
Cav-1 promotes U251 glioma cell proliferation *in vitro*. U251 cells were transfected with empty vector pEGFP-C3 or the vectors expressing Cav-1, siCav-1, or negative control siRNA (siNC). The transfection efficiency was confirmed by qPCR (**A**) and western blot analysis (**B**). **C**, Cell proliferation was measured at time points as indicated using the CCK-8 assay. **D**, Human umbilical vein endothelial cells (HUVECs) and U251 glioma cells were transfected with pEGFP-C3, pEGFP-C3-Cav-1, siCav-1, or siNC and verified by enhanced chemiluminescence assay. Images were captured at magnification 40 (scale bar 500 μm) and 100× (scale bar 100 μm). Representative images are shown. **E**, Branch points in 3 randomly selected fields were counted using ImageJ software. Data are reported as means±SE. **P<0.01, ***P<0.001, ****P<0.0001 *vs* other groups (transfected with pEGFP-C3, siCav-1, or siNC; ANOVA) for n=3. NC: negative control.

To determine whether Cav-1 functions in glioma development and progression, tumor cell proliferation, HUVEC tubule formation, and VM formation were examined using vectors expressing Cav-1 or siCav-1 (see Supplementary Figure S1) to overexpress or knock-down Cav-1 in U251 glioma cells, respectively. The transfection efficiency is shown in Figure 3A and B. We found that Cav-1 overexpression significantly promoted while Cav-1 knockdown significantly inhibited U251 cell proliferation at 48 and 72 h after transfection compared with the control groups (untreated and siNC-transfected cells) (Figure 3C). In addition, 3D culture showed that the conditioned medium from Cav-1 overexpressing-U251 cells significantly increased tubule formation in HUVECs compared with the control groups. Cav-1 overexpression also significantly promoted VM formation in U251 cells. Opposite effects were observed in Cav-1-deficient U251 cells (Figure 3D and E). Taken together, these results demonstrated that Cav-1 promoted tumor cell proliferation and vascular formation in glioma, contributing to glioma development and progression.

### Cav-1 regulated the expression of AKT and HIF-1α

**Figure 4:**
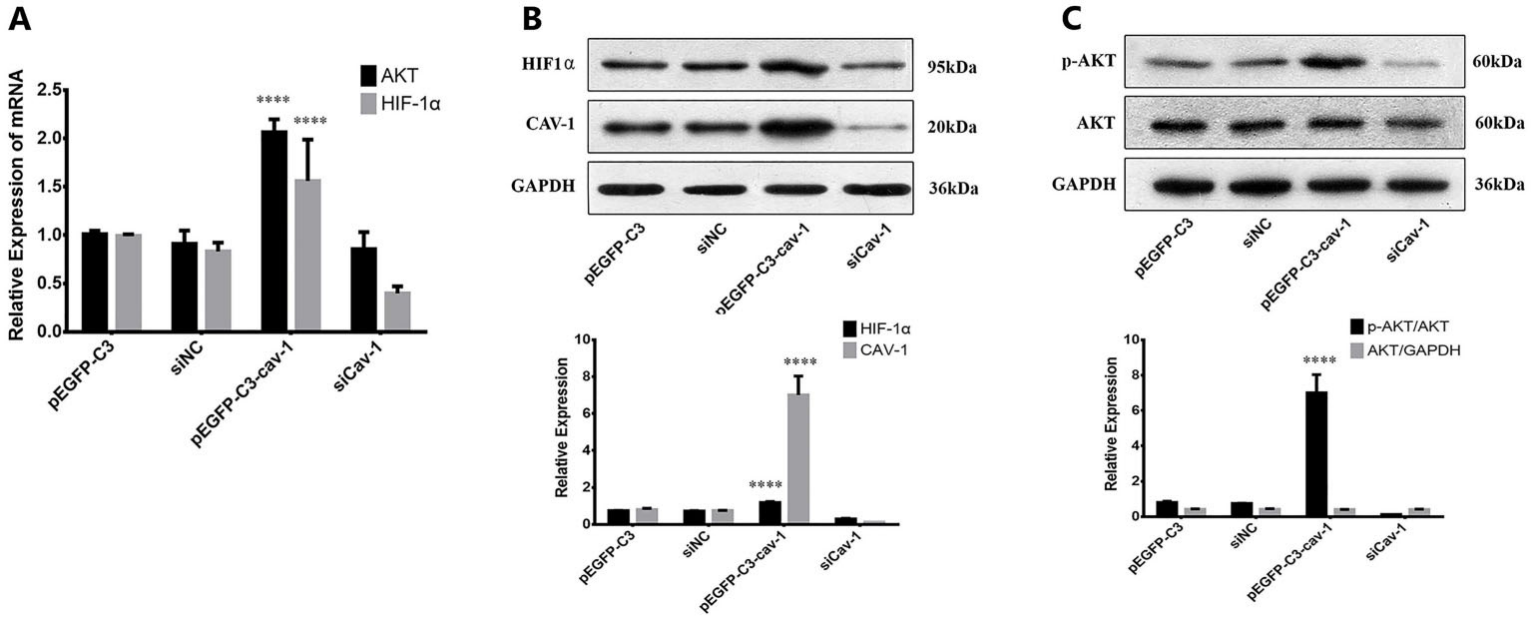
Cav-1 regulates p-Akt and HIF-1α expression. U251 cells were transfected with pEGFP-C3, pEGFP-C3-Cav-1, siCav-1, or siNC. The mRNA expressions of Akt and HIF-1α were determined by qPCR (**A**) and protein levels of HIF-1α (**B**), and Akt and p-Akt (**C**) were assessed by western blot analysis. ****P<0.0001 *vs* other groups (transfected with pEGFP-C3, siCav-1, or siNC; ANOVA) for n=3. NC: negative control.

To investigate the molecular mechanism underlying the promotive role of Cav-1 in VM formation in glioma, qPCR and western blot analysis were performed to examine the expression of VM formation-associated Akt and HIF-1α (18). Both mRNA and protein levels of Akt and HIF-1α were elevated by Cav-1 overexpression but suppressed by siCav-1 in U251 cells (Figure 4A and B), and p-Akt level was significantly increased by Cav-1 overexpression (Figure 4C), suggesting that activated Akt signal pathway may be involved in Cav-1-induced glioma cell proliferation and VM formation.

## Discussion

VM is a non-endothelium-dependent vasculature that complements the endothelium-dependent vessels in providing oxygen and nutrients for malignant tumor cells. Although it has been widely investigated in the pathology of glioma, which is characterized by increased microvasculature in tumor tissues (26,27), the molecular mechanism governing VM formation in glioma remains largely unknown. Our previous study has shown that Cav-1 and HIF-1α play important roles in the progress of glioma, and both of them are significantly associated with glioma prognosis (28). Considering that Cav-1 contributes to VM formation through the PI3K/Akt signaling cascade in uveal melanomas (21), we hypothesized that there might be an association between Cav-1 and VM in glioma.

In the present study, Cav-1 expression and VM formation were examined by immunohistochemistry and CD31/PAS double-staining, respectively, in human glioma tissues. The results demonstrated the presence of VM in glioma, which is consistent with previous studies (29,30). We also found that Cav-1 expression and VM formation were significantly upregulated in HGG compared with LGG, and both of them were significantly correlated with shortened survival of glioma patients, suggesting Cav-1 or VM formation as a prognostic indicator in glioma. These findings are consistent with previous research demonstrating that patients with VM-positive gliomas survived a shorter period of time than those with VM-negative gliomas (25), suggesting that VM formation is accompanied by increasing malignancy and higher aggressiveness. Therefore, targeting VM is a promising therapeutic strategy for glioma therapy. Since Cav-1 upregulation and VM formation are both correlated with glioma grades and outcomes, we next sought to investigate whether there is a correlation between Cav-1 and VM. Indeed, VM formation was found positively correlated with Cav-1 expression in glioma tissues, and patients with Cav-1 overexpression and increased VM formation in combination had poorer prognoses than other patients, suggesting a synergistic role of Cav-1 and VM in glioma progression. To elucidate the causal relationship between Cav-1 and VM, gain- and loss-of-function analyses were conducted in U251 glioma cells. The results showed that Cav-1 is essential for U251 cell proliferation and VM formation, which is in agreement with the findings in uveal melanoma (21).

Hypoxia is a universal feature of growing solid tumors because of their high demands for oxygen and nutrients caused by cell proliferation (31). Previous studies have shown that hypoxia induces HIF-1α and HIF-2α, which stimulate the formation of tumor vasculatures, including angiogenesis and VM formation, through vascular endothelial growth factors (32). In this study, to further understand the effect of Cav-1 on VM formation, the expression level of HIF-1α was evaluated. We found that Cav-1 was an upstream regulator governing the expression of HIF-1α in glioma cells, which suggests a promotive role of Cav-1/HIF-1α/VM axis in glioma development. These data, together with a previous finding demonstrating that hypoxia induces HIF-1α-dependent upregulation of Cav-1 expression in hepatocellular carcinoma (23), suggest a functional interplay between Cav-1 and HIF-1α in tumors in the event of hypoxia. Further investigation is needed for more confirmatory tests and to explore the downstream signaling pathway of this interplay.

Moreover, based on the findings that Cav-1 expression is correlated with PI3K activity and VM formation in primary uveal melanoma tissues (21), the expression of Akt, the major downstream effector of PI3K (33), was evaluated. Guo et al. reported that the AKT pathway is critically involved in hypoxia-induced VM formation in glioma cells (34). Our results showed the level of p-Akt was significantly elevated by Cav-1 overexpression but suppressed by siCav-1 in U251 cells, suggesting that activated Akt signal pathway may be involved in Cav-1-induced glioma cell proliferation and VM formation. However, more experiments, like knockdown or inhibition of Akt or HIF-1α in Cav-1-overexpressing glioma cells and protein-protein interactions in hypoxic conditions, need to be done to evaluate the direct role of Akt and HIF-1α in glioma VM formation.

In conclusion, our study suggests that Cav-1 expression and VM formation may be correlated with each other and both of them could be unfavorable prognostic factors in patients with glioma. Cav-1-dependent expression of HIF-1α and Akt may be involved in the promotive role of Cav-1 in glioma cell proliferation and VM formation. Thus, anti-VM therapies should focus on Cav-1 or its downstream VM-associated genes to develop more effective drugs to treat glioma.

## Supplementary Material

Click here to view [pdf].
